# Clinical Utility of Liquid Biopsy-Based Actionable Mutations Detected via ddPCR

**DOI:** 10.3390/biomedicines9080906

**Published:** 2021-07-28

**Authors:** Irina Palacín-Aliana, Noemí García-Romero, Adrià Asensi-Puig, Josefa Carrión-Navarro, Víctor González-Rumayor, Ángel Ayuso-Sacido

**Affiliations:** 1 Atrys Health, 08025 Barcelona, Spain; ipalacin@atryshealth.com (I.P.-A.); aasensi@atryshealth.com (A.A.-P.); vgrumayor@atryshealth.com (V.G.-R.); 2 Fundación de Investigación HM Hospitales, HM Hospitales, 28015 Madrid, Spain; 3 Faculty of Science, Universidad de Alcalá, 28801 Madrid, Spain; 4 Faculty of Experimental Sciences, Universidad Francisco de Vitoria, 28223 Madrid, Spain; noemi.garcia@ufv.es (N.G.-R.); pepa.carrion@ufv.es (J.C.-N.); 5 Brain Tumor Laboratory, Fundación Vithas, Grupo Hospitales Vithas, 28043 Madrid, Spain; 6 Faculty of Medicine, Universidad Francisco de Vitoria, 28223 Madrid, Spain

**Keywords:** liquid biopsy, ddPCR, cancer biomarkers, lung cancer, breast cancer, colorectal cancer, pancreatic cancer

## Abstract

Cancer is one of the leading causes of death worldwide and remains a major public health challenge. The introduction of more sensitive and powerful technologies has permitted the appearance of new tumor-specific molecular aberrations with a significant cancer management improvement. Therefore, molecular pathology profiling has become fundamental not only to guide tumor diagnosis and prognosis but also to assist with therapeutic decisions in daily practice. Although tumor biopsies continue to be mandatory in cancer diagnosis and classification, several studies have demonstrated that liquid biopsies could be used as a potential tool for the detection of cancer-specific biomarkers. One of the main advantages is that circulating free DNA (cfDNA) provides information about intra-tumoral heterogeneity, reflecting dynamic changes in tumor burden. This minimally invasive tool has become an accurate and reliable instrument for monitoring cancer genetics. However, implementing liquid biopsies across the clinical practice is still ongoing. The main challenge is to detect genomic alterations at low allele fractions. Droplet digital PCR (ddPCR) is a powerful approach that can overcome this issue due to its high sensitivity and specificity. Here we explore the real-world clinical utility of the liquid biopsy ddPCR assays in the most diagnosed cancer subtypes.

## 1. Introduction

### 1.1. Background

Despite many advances in the field, cancer remains one of the primary causes of death worldwide. In fact, estimations point to a total of 19.3 million new cases and almost 10 million deaths per year with no distinction between developed and undeveloped countries [1]. The most diagnosed cancer type worldwide is breast cancer followed by prostate cancer. On the other hand, lung cancer shows the highest mortality with an estimated 1,796,144 deaths worldwide in 2020 [2]. According to these statistics, it is not surprising that cancer has become the mainstay of research all over the world. In the last few years, many efforts in this field have been made, majorly in the diagnosis and prognosis of the different types of cancer, to assist clinicians to make more effective treatment selections through novel technologies. Nowadays, tumor tissue is the main source of information for diagnosis, stratification, prognosis, and treatment decision, discriminating between cancer or non-cancer lesions and providing significant information of different clinically relevant biomarkers [3,4,5]. 

**Figure 1:**
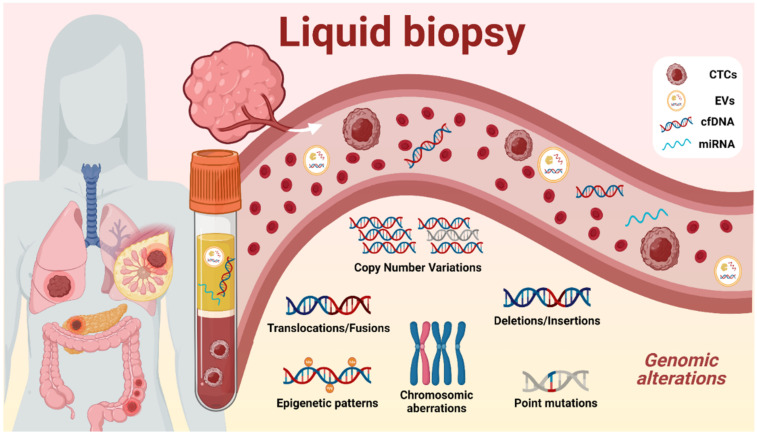
Schematic of the liquid biopsy composition. Liquid biopsy obtained from peripheral blood is composed of different tumoral components such as circulating tumor cells (CTCs), circulating cell-free DNA (cfDNA), extracellular vesicles (EVs), and micro-RNA (miRNA). These elements can be isolated for the identification of various tumor-specific genomic aberrations including point mutations, copy number variations, structural rearrangements, or epigenetic patterns.

Although tissue biopsies are crucial in the current evaluation and classification of cancer types, they present many limitations. Some of these relate to the highly invasive techniques required to obtain the biopsies and the risk to the patient associated with these procedures, as some tumors are hardly accessible due to their anatomical location and/or their infiltrative nature [5]. Also, tissue biopsies fail to represent the intra-tumoral heterogeneity since only a small fraction of the tumor obtained will be evaluated by physicians. To overcome this issue, liquid biopsies are present as a new minimally invasive tool for intra-tumoral monitoring which represents more accurately the tumoral status. Samples are collected from different biofluids, mostly from blood (serum and plasma) but also from saliva, breast milk, cerebrospinal fluid, stool, semen, urine, etc. [6]. Several biomarkers represented in Figure 1 could be isolated from different sources such as circulating tumor cells (CTCs), extracellular vesicles (EVs), cell-free DNA (cfDNA), and micro-RNA (miRNA).

The circulating DNA found in all biofluids is referred to as cfDNA, which includes tumor-derived fraction called circulating tumor DNA (ctDNA) [7]. Nevertheless, cfDNA has been proven to be a powerful tool since all tumor cells, indistinctly of their phenotype, secrete DNA into the biofluids, providing information of the whole tumor, and revealing a snapshot of the intra-tumoral heterogeneity state at the moment of sample collection [8]. The mostly minimal-invasive nature of liquid biopsies allows clinicians to be informed about the molecular evolution of the tumor genetics, permitting disease monitoring, and avoiding the classical biopsies that could endanger the patient. The amount and length of ctDNA has been investigated due to its potential capacity for early detection and prognosis of some tumors. Also, in the current state of the art, ctDNA techniques are capable of detecting the genomic aberrations represented in Figure 1, such as copy number variations (CNVs), methylation changes in DNA promoters, and single-nucleotide variations (SNVs) in a biofluidic sample [9], giving remarkable information about treatment response, tumoral staging, prognosis, minimal residual disease, and actionable mutations, enhancing more precise clinical decisions.

### 1.2. Droplet Digital PCR (ddPCR)

Nowadays, there are many technologies that allow for the detection of the ctDNA fraction [10]. Although the traditional PCR-based assays are the cheapest ones, they have limited sensitivity and specificity [11]. Due to the novel mutational status information provided in one single assay by the next-generation sequencing (NGS) technique, their sensitivity and specificity have increased to 0.1% [11,12]. On the downside, NGS is expensive, highly time-consuming, and requires well-experienced bioinformaticians to discriminate between actionable tumoral mutations and normal tissue background [13]. In this context, droplet digital PCR (ddPCR) has newly emerged as a powerful and cost-effective tool [14], capable of the detection and absolute quantification of point mutations up to 0.01% with no need for specific bioinformatic interpretation (Table 1) [15,16]. 

**Figure 2:**
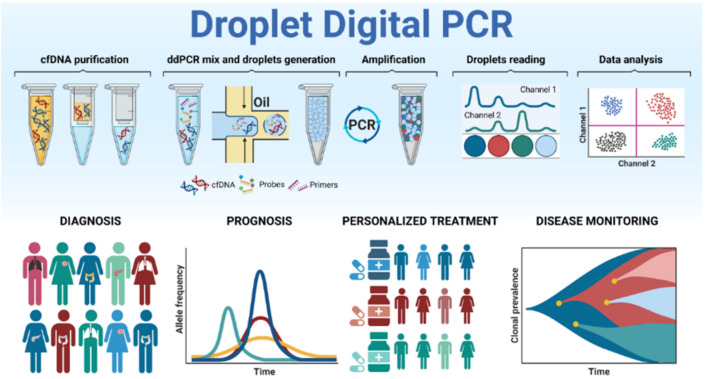
Summary of the ddPCR alterations screening process. The purified cfDNA is divided into thousands of oil droplets together with specific primers and probes. The ddPCR currently has several applications such as cancer diagnosis, prognosis, personalized treatment administration, and disease monitoring.

As shown in Figure 2, ddPCR divides the nucleic acids into thousands of individual end-point PCR reactions permitting their single analysis via oil sphere microfluidics. In oncology, ddPCR is further being used not only for point mutations detection and absolute quantification but is also currently employed for rare mutation detection, CNVs, DNA methylation, and gene rearrangements screening in different sources of clinical samples [14,22].

The near future of cancer diagnosis, prognosis, and treatment is expected to explore liquid biopsy biomarkers as an additional test to guide clinicians in every step of disease management. In this sense, an increasing number of cancer-derived biomarkers are being identified. In this review, we unify and classify different acknowledged and potentially actionable mutations that are relevant in lung, breast, colorectal, and pancreatic tumors, for further detection by ddPCR in clinical laboratories, in order to ease the way for clinicians in every step of disease management, striving to advance efforts in cancer diagnosis, prognosis, and treatment. To the best of our knowledge, this is the first report that presents a compendium of all the ongoing liquid biopsy-ddPCR clinical trials.

## 2. Lung Cancer

Despite all the advances made in the past years, lung cancer remains the leading cause of neoplastic death worldwide [1]. The gold standard for non-small cell lung cancer (NSCLC) genotyping is the analysis of different driver genes such as *EGFR*, *BRAF*, *HER2*, *KRAS*, and *MET*, as well as rearrangements in *ALK*, *ROS1*, *RET*, and *NTRK1/2/3* [23,24], typically analyzed by PCR-based assays, immunohistochemistry (IHC), and fluorescent in situ hybridization assays (FISH) [25]. The introduction of genetic studies has significantly improved targeted therapies and subsequently patient’s progression-free survival (PFS) and overall survival (OS). Some of the successful treatments involve immunotherapies, such as the inhibition of the PD-1/PD-L1 axis [26]. ddPCR was also used for these immune targeted markers in solid [27] and liquid biopsies [28].

Routinely, the testing for molecular tumor alterations is performed on resected tissue biopsies. However, in some situations, when there is insufficient material for molecular analysis or the patient is unfit for invasive tissue sampling, the analysis of cfDNA has already been introduced [29].

### 2.1. EGFR

The epidermal growth factor receptor, known as *EGFR*, is one of the most characterized genes in lung cancer and appears in around 10–35% of NSCLC patients [30]. *EGFR* mutations (exon 21 L858R and L861Q or *EGFR* exon 19 deletions) represent 85% of EGFR mutations [24,30,31], which confer sensitivity to *EGFR* tyrosine kinase inhibitors (TKIs) [23], and could be detected by multiplexed ddPCR assays with a sensitivity of at least 0.20% [32]. The most common TKIs used in clinical practice are erlotinib, gefitinib, and neratinib [33]. Guidelines recommend *EGFR* mutation genotyping to guide personal therapy by identifying NSCLC patients that may benefit EGFR-TKIs [34]. Unfortunately, after 8–14 months of first-line TKIs treatment, most of the patients acquired therapy resistance resulting in disease progression or relapse. This acquired resistance is caused mainly by the appearance of the T790M mutation in 60% of the NSCLC patients [30,31]. Therefore, its detection by ddPCR has been implemented in several hospitals and a wide set of studies highlights its importance. In a large cohort of 343 NSCLC patients, *EGFR* T790M was detected in 24% of patients by ddPCR [35]. These data were further corroborated in a smaller cohort of patients in progression, in which they detected 52% of positive samples with a cfDNA frequency of 0.5% [36].

For the treatment of those patients who acquired first-generation resistance, a third-generation TKI therapy, osimertinib, has been developed. Different studies quantified the mutation allele frequency (MAF) of the T790M mutation before and during the course of osimertinib treatment [34,37]. A retrospective study observed how patients with partial response or stable disease to second-line therapy with osimertinib had higher T790M mutant frequency in plasma cfDNA than those with progressive disease. Although higher T790M MAF levels were associated with longer PFS and OS [38], Li et al. didn’t report differences in response rate or PFS and OS [39]. Additional studies are needed for the standardization of the MAF quantification cutoff value and to assess whether the quantitative measurements of plasma cfDNA T790M mutation could be used to predict TKIs therapy response.

Inevitably, novel acquired *EGFR* mutations conferring third-generation TKI osimertinib resistance have been observed. Approximately 20–40% of these cases are caused by the C797S mutation, which avoids drug covalent binding [40]. Via ddPCR, *EGFR* T790M and C797S mutations have been longitudinally assessed in plasma cfDNA during and after treatment [41]. A recent report showed three molecular patterns based on the presence/absence of T790M, C797S, and Ex19Del mutations, which could help in clinical decisions [40]. Serial evaluation of different *EGFR* mutations in plasma cfDNA during osimertinib treatment may be useful as a prognostic factor for disease progression. More importantly, those patients with T790M mutation clearance and detectable levels of C797S resistance mutation together with a sensitizing *EGFR* mutation may benefit from first-generation TKIs re-treatment [42,43].

Apart from the most scrutinized mutations in *EGFR*, some researchers are focused on the development of ddPCR assays for less common mutations, such as G719S and L851Q, in advanced NSCLC patients [44].

The detection of *EGFR* mutations has been explored in other liquid biopsies as the bronchial washing fluid (BWF) [45,46], the fine-needle aspiration (FNA) supernatants, sputum, and urine [47,48,49]. Lastly, for lung cancer patients that develop metastases to the central nervous system (CNS), detection of *EGFR* mutations in the cerebrospinal fluid (CSF) has been proven to be more efficient than plasma to evaluate PFS [50].

### 2.2. KRAS

*KRAS* is an oncogenic driver gene that appears to be mutated in 25–30% of NSCLC patients [51]. Mutations in codons 12 and 13 of this gene are the most frequent alterations and stand as the principal cause of the development and progression of several cancer types [52]. Despite all clinical advances in personalized therapies and their proven impact on patient’s clinical outcome, there is only one effective drug (sotorasib) [53] approved by the Food and Drug Administration (FDA) for *KRAS* G12C NSCLC patients [54,55].

It has been suggested that *KRAS* mutations decrease EGFR-TKIs sensitivity through the MAPK/ERK pathway activation in NSCLC patients [53,56]. Moreover, the presence of *KRAS* mutations is also related to high PD-L1 levels, suggesting that those patients could be good responders to immune checkpoint inhibitors [57]. In contrast, *KRAS* mutations appear to decrease the anti-angiogenic bevacizumab effects [58].

The design of ddPCR multiplex assays to detect the most frequent G12/G13 *KRAS* mutations has allowed for rapid and accurate genotyping of plasma cfDNA with a LOD of at least 0.2% [51,52,59,60]. As a result of these assays, an association between *KRAS* mutated concentration and disease stage has been observed. Patients with advanced lung cancer stages present greater amounts of detectable *KRAS* mutations in plasma cfDNA samples, being 8% in stage I, 30% in stage II, 71% in stage III, and 73% in stage IV [51]. Furthermore, it has been observed that patients with stable disease presented lower *KRAS* mutations levels than patients who had progressed [52]. Also, several studies have associated plasma ctDNA *KRAS* mutations with shorter PFS and OS [51,52,59,60], and chemotherapy treatment efficacy has been longitudinally monitored, showing a connection between ctDNA *KRAS* concentration changes and therapy response [51,61,62]. 

Despite being a limited druggable target mutation, the analysis of ctDNA *KRAS* mutations could be used as a prognostic and predictive tool and may guide alternative therapy approaches such as chemotherapy or immunotherapy. 

### 2.3. ALK

The tyrosine kinase receptor *ALK* is found rearranged, mostly with Echinoderm Microtubule-Associated Protein-like 4 (*EMLA4*), in approximately 5% of NSCLC patients, causing inappropriate signaling which induces an activated state in cancer cells [63]. The gold standard, with a LOD of 15%, is its direct visualization by FISH or IHC [64]. Conversely, NGS has been recently recommended for the identification of different NSCLC rearrangements, including *ALK* [65]. As a more sensitive technique, a ddPCR assay has been designed to detect *ALK-EMLA4* gene translocations with a LOD of 0.25% in formalin-fixed paraffin-embedded (FFPE) samples [64].

Patients presenting *ALK-EML4* rearrangements appear to be sensitive to the ALK-TKIs crizotinib, ceritinib, alectinib, brigatinib, and lorlatinib [29,66,67]. Approximately 20% of patients with *ALK* rearrangements treated with first-generation ALK-TKI crizotinib develop resistance due to mutations in the kinase domain [68]. Although the most common and relevant *ALK* resistance mutations are the G1202R and F1174C/L, there are more than 10 mutations described which confer resistance to first-generation ALK-TKIs, such as L1196M, G1269A, C1156Y, 1151Tins, L1152R, S1206Y, I1171T, D1203N, and V1180L (Table 2) [67,69]. For those patients who develop resistance, second-generation ALK-TKIs ceritinib, alectinib, and brigatinib have been developed [68]. Unfortunately, G1202R mutation confers resistance to second-generation ALK-TKIs in some of the patients [70], which could be treated with lorlatinib (a third-generation ALK-TKI inhibitor). Three ddPCR multiplexed assays are available to specifically detect the 10 previously detailed *ALK* mutations. In a small cohort of 7 *ALK*-positive NSCLC, the monitoring of the different *ALK* resistance mutations status during the course of the disease was successfully performed [71] but further and wider studies are required to assess the ddPCR clinical utility to detect *ALK* secondary mutations and their implication in patients PFS and OS. In any case, these assays offer a fast and sensitive technique for the monitoring of newly discovered resistance mutations by minimally invasive cfDNA liquid biopsies. 

## 3. Breast Cancer

Breast cancer (BC) has recently surpassed lung cancer as the most lethal and diagnosed neoplasm in women [1]. Routinely for clinical practice, BC is classified into five subtypes based on histological and molecular characteristics [72]. Tumors expressing estrogen receptor (ER) and/or progesterone receptor (PR) are considered hormone receptor (HR)-positive BC and those expressing the human epidermal growth factor receptor 2 (HER2 or ERBB2) are diagnosed as HER2-positive BC. Samples with no ER, PR, nor HER2 expression, are classified as triple-negative breast cancer (TNBC) [72]. These markers are used to guide personalized treatment administration and to predict responses to endocrine and immune therapy.

Novel agents, which effectiveness depends on specific genomic aberrations, are being developed. The most common targets are *PIK3CA*, *HER2*, androgen receptor (*AR*), *AKT1*, *ESR1,* and PD-L1 [73]. The evaluation of the tumor genomic alterations in plasma cfDNA has been largely evaluated especially in metastatic BC (mBC) patients due to the high levels of ctDNA released into the bloodstream. The appearance of more sensitive technology than Sanger sequencing such as ddPCR has confirmed the presence of ctDNA before and after surgery in early-stage BC patients [74], in which it can predict tumor recurrence even 11 months earlier than traditional methods [75,76]. The assessment of cfDNA has great benefits for therapy guidance and a prognostic value in all BC disease stages.

### 3.1. BRCA

A 10% of BC cases are hereditary and associated with family clinical history [72]. The *BRCA* family genes are the most frequently mutated in BC, whose aberrations increase the risk of developing BC up to 70% [72]. Patients harboring *BRCA* alterations may benefit of poly (ADP-ribose) polymerase (PARP) inhibitors increasing PFS, OS and their quality of life [72]. 

A wide spread of *BRCA* gene alterations have been described, such as point mutations, large genomic rearrangements or CNVs. Sanger sequencing and Multiplex ligation-dependent probe amplification (MLPA) were thought to be the most reliable methods [77]. Despite excellent concordance rate between MLPA and ddPCR in the detection of *BRCA1* genomic rearrangements [78], it is six times more expensive and requires at least one separate reaction for each gene exon. In this sense, a more cost-effective ddPCR based on an amplitude multiplex has been developed covering all coding and non-coding exons, together with two reference genes (*RPP30* and *ALB*) [79]. 

Even though ddPCR can be useful for genotyping *BRCA* genes in liquid biopsies, further optimization and standardization in larger cohorts is needed to clarify its clinical application and significance. 

### 3.2. PIK3CA

Hormonal therapies have greatly improved ER-positive mBC patient outcomes. Unfortunately, *PIK3CA* exon 9 E545K and E542K, and *PIK3CA* exon 20 H1047R, and H1047L mutations are frequently associated with resistance to hormonal therapies, such as fulvestrant [72,80]. For this reason, the identification of those biomarkers in plasma ctDNA could be used to predict BC treatment. In fact, a relationship has been shown between these mutations and a good response to anti-PI3K and anti-CDK4/6 targeted therapies together with fulvestrant in HR-positive HER2-negative advanced BC [81,82].

ddPCR was used to analyze *PIK3CA* ctDNA mutations in the PALOMA-3 (NCT01942135), MIRHO (NCT01612871), and BOLERO-2 (NCT00863655) clinical trials. Although PALOMA-3 and MIRHO trials did not report an association between *PIK3CA* baseline status and PFS, it has been shown that BC *PIK3CA*-mutated patients treated with a combination of palbociclib and fulvestrant improves PFS [80,82]. In contrast, the mutational analysis from patients enrolled in the BOLERO-2 trial receiving second-line treatment with everolimus, an mTOR1 inhibitor, showed that *PIK3CA* mutations had no effect on its effectiveness and that they are not a predictive determinant for everolimus benefit [83].

It has been widely proven that early identification of the mBC ctDNA *PIK3CA* mutation status, could allow future evaluation of disease response or progression and eventually, better treatment administration.

### 3.3. ESR1

Another interesting gene to point out is the *ESR1* ligand-binding domain *(LBD)*, which is mutated in 30–40% of ER-positive mBC patients [84,85]. Importantly, a significant number of mBC patients treated with first-line aromatase inhibitors (AIs) acquire *ESR1* LBD mutations during the treatment developing endocrine treatment resistance [84,86,87]. Through ddPCR, several researchers have analyzed the *ESR1* mutational status in peripheral blood [86,88,89]. The presence of *ESR1* Y537S and D538G mutations in ER-positive mBC has been observed prior to treatment administration [90]. A significant increase in mutation prevalence was observed in patients who already received first-line AI therapy compared with those patients who only received adjuvant AI therapy [90]. Further analysis demonstrated the association of Y537S and D538G *ESR1* mutations with worse OS. 

The clinical significance of monitoring *ESR1* LBD mutations Y537S, Y537N, Y537C, and D538G has been assessed in a cohort of sequential plasma samples from mBC and ER-positive primary BC treated with different endocrine therapies [91]. ddPCR data presented the ctDNA *ESR1* mutation fluctuation as a consequence of treatment and showed that increasing amounts of *ESR1* mutation post-therapy resulted in a poor response to treatment [91]. 

Various multiplexed assays have been developed to easily and simultaneously monitor different hotspot mutations in the *ESR1* LBD gene. The decrease of *ESR1* Y537S, Y537N, and D538G plasma detection in ER-positive BC women increase PFS and therapy effectiveness [84]. This impact has also been observed using another multiplex assay, in which eight different mutations; E380Q, L536H, L536R, Y537C, Y537N (T > A), Y537N (delinsTA), Y537S, and D538G were studied with high sensitivity and a LOD of 0.07–0.19% [92]. 

Even though *ESR1* mutations have been principally detected in mBC, the ddPCR implementation allows detection of *ESR1* mutations in approximately 2.5–7% of primary BC [93]. Furthermore, it has been observed that *ESR1* mutations can be more frequently detected in cfDNA than in tissue biopsies [88].

### 3.4. HER2

*HER2* is amplified in approximately 20–30% of invasive BC patients. HER2 overexpression causes tumor cell proliferation, aggressiveness, and subsequently, poor prognosis [72]. Nowadays, the combination of monoclonal antibodies trastuzumab or pertuzumab with chemotherapy has improved the PFS and OS in patients with early-stage and metastatic BC (Table 3) [94]. Despite the benefit of this combined therapy, some patients do not respond to treatment administration, resulting in poor survival.

The gold standard approach to assess *HER2* amplification in tissue samples is IHC or FISH [72]. Although little is known about the *HER2* amplification detection in liquid biopsies, a ddPCR assay has been optimized to detect its CNVs in plasma [95]. To ensure proper detection, the gene *EFTUD2* (elongation factor Tu GTP-binding domain 2) was used as a reference. The designed HER2:EFTUD2 ddPCR assay showed a high concordance of 90% with matched tumor biopsies [95], 100% sensitivity, and 98% specificity in a cohort of 76 BC patients [96]. Even though the protocol has been principally developed for the detection of plasma-derived cfDNA it can be adapted for FFPE or fresh frozen tissue samples [97].

These minimally invasive tests that identify *HER2* amplification would be a clinical turnaround. Additionally, this approach could be modified for the evaluation of any amplified gene in cancer. Especially, it might be a beneficial approach for unusual acquisition events in response to therapy. Future prospective studies with larger cohorts should be conducted to evaluate these potential biomarkers and to optimize the ddPCR assays.

### 3.5. ddPCR Assays for Multiplex Genes

ddPCR multiplexed assays have been introduced as procedures to reduce the number of reactions and the sample volume employed. A prospective study developed eight optimized multiplexed ddPCR assays for 20 targetable hotspot mutations in the *PIK3CA* (E545K, H1047L, H1047R, and E542K), *ESR1* (Y537C, Y537N, Y537S, V534E, S463P, L536Q, E380Q, D538G, and L536R), *AKT1* (E17K), and *HER2* (L869R, L755S, V777L, S310F, D769H, and L755_759del). Data were compared with NGS results, revealing an excellent concordance of 79.5%. Since the NGS technique is not as sensitive as ddPCR, the major reason for the discordant cases were mutations detected by ddPCR and undetectable with NGS [73]. 

A similar multiplexed assay has been used for the evaluation of the possible effects of delayed plasma processing. For that, paired blood samples were processed immediately for 48–72 h after collection, in which the agreement in mutation screening was as high as 94.8% [98]. 

### 3.6. Other

BC cancer stem cells (BCSCs) are being investigated as a novel therapeutic approach to identify early genetic alterations in tumor evolution [99]. In a prospective study, plasma cfDNA from patients with early-stage and advanced BC were used to detect, through ddPCR, previously studied BCSC gene mutations [100]. BCSC ribosomal protein L39 (RPL39), A14V, and myeloid leukemia factor 2 (MLF2) R158W mutations were detected in 29% of cfDNA samples from early-stage BC patients and in 40% of mBC. The presence of any of the mutations was associated with significantly worse OS and interestingly, the increasing BCSCs gene mutation detection in the patients’ plasma cfDNA highly correlated with the disease stage. 

## 4. Colon and Rectal Cancers

Colorectal Cancer (CRC) follows lung and breast cancers as the third neoplasm cause of death [1]. Due to the delayed start of symptoms, less than 40% of the patients are diagnosed with early-stage or localized disease. Consequently, the majority of patients are diagnosed with advanced localized disease and/or distant metastases, both with a high risk of recurrence after surgical resection [101]. 

CRC is known to be initiated by an accumulation of several mutations in a subset of crucial genes involved in the regulatory pathways. These genes, such as *APC*, *KRAS*, *BRAF*, *PIK3CA,* and *SMAD4,* are highly implicated in cellular replication, proliferation, and invasiveness [101]. Since CRC release elevated quantities of DNA into the bloodstream, several studies have described the potential use of plasma cfDNA in the diagnosis, management of patients, and as a tumor recurrence marker (Table 4) [102]. 

### 4.1. KRAS and BRAF

Over the last decade, most therapies have been designed to target aberrant signaling or activation of the MAPK pathway, such as the overexpression of *EGFR* found in 50–80% of patients [113,117,130]. Unfortunately, *KRAS* or *BRAF* mutations trigger non-responsiveness to anti-*EGFR* monoclonal antibody therapies [115,116,117]. *KRAS* mutations G12/G13 are observed in about 35–45% of CRC patients [111,117] and the first-line of treatment relies on a combination of chemotherapy of fluoropyrimidine with oxaliplatin or irinotecan [111]. *BRAF* shows lower mutation rates and appears in about 8–12% of metastatic CRC (mCRC), with V600E being the most common [118]. Although, the use of *BRAF* inhibitors alone such as vemurafenib did not reach the expected effectiveness, the combination with anti-*EGFR* monoclonal antibodies, MEK, and/or PI3K inhibitors have shown promising outcomes [118]. 

Different PCR-based platforms (Bio-Rad ddPCR, BioCartis Idylla, Roche COBAS z480, and Sysmex BEAMing) have been tested for the detection of plasma ctDNA *KRAS* mutations. Among the four platforms, ddPCR and BEAMing resulted to be the most sensitive techniques. In addition, ddPCR and COBAS were the ones that allowed the analysis of a higher number of samples per reaction [103]. Another retrospective study used mCRC plasma ctDNA to compare COLD-PCR, a microarray-based approach, and ddPCR. As expected, ddPCR showed the highest concordance in the identification of ctDNA mutations previously genotyped on tissue samples [107]. Furthermore, ddPCR was demonstrated to be the faster protocol, the most cost-effective method with the higher sample throughput setup, and the most suitable and replicable technology to assess the tumor genotype using liquid biopsies [107,108].

The DECALIB study was one of the first prospective studies to use the ddPCR technique to evaluate and compare the early detection of *KRAS* and *BRAF* mutations present in plasma cfDNA and tissue [109]. Afterwards, many other studies have also evaluated the concordance between tissue and plasma *KRAS* mutations obtaining elevated specificity and sensitivity rates [102,110]. Even though many different manuscripts have observed better correlations between cfDNA concentrations and the tumor mutation burden in mCRC [107,110,131], the cfDNA analysis could be also performed in patients at earlier cancer stages [102,110].

Longitudinal analysis of circulating *KRAS* concentrations in mCRC has great prognostic value since it has the ability of outcome prediction and treatment monitoring. In a *KRAS*-positive mCRC cohort from the prospective multicenter AIO KRK0207 trial (NCT00973609), ctDNA *KRAS* mutations were quantified via ddPCR before and 2–3 weeks after first-line chemotherapy initiation with fluoropyrimidine, oxaliplatin, and bevacizumab. Individuals with ctDNA *KRAS* mutations detected at baseline and in follow-up measurements presented worse OS and PFS. Remarkably, *KRAS* mutations identified at baseline in 15% of the patients were not detectable at follow-up measurements after treatment initiation. Those patients with *KRAS* mutation clearance at follow-up had better disease control and most notably better OS and PFS [111]. The same results have been observed by Holm et al. in patients included in the AXOAXI trial (NCT01531595 and EudraCT 2011-003137-33), treated with bevacizumab in combination with altering capecitabine and oxaliplatin or irinotecan [132], or by Kelin-Scory et al. where *KRAS* mutations cleared precipitously independently of type and intensity of chemotherapy and regardless of bevacizumab anti-*VEGF* treatment [112]. *KRAS* mutations have been detected 10 months earlier than radiographic confirmation of disease progression [113]. More remarkably, these ctDNA *KRAS* fluctuations and final disappearance open the possibility and potential treatment effectiveness of anti-*EGFR* therapies in those CRC patients [112,114].

Alternative sources of DNA such as EVs and fluids in the surrounding area of the tumoral tissue have been shown to provide information about disease evolution [104,133]. Fluids in the surrounding tumoral area such as the peritoneal fluid have been shown to be useful liquid biopsy sources of cfDNA [104]. In CRC patients with peritoneal metastases, a significantly higher amount of *KRAS* or *BRAF* ctDNA has been observed in peritoneal fluid than in plasma [105]. Since urine collection can be easily and repeatedly self-performed at any location with minimal effort, it has also been presented as an alternative source of cancer biomarkers for disease progression and drug response monitoring. In a proof-of-concept study with a *KRAS*-positive mCRC cohort, *KRAS* or *BRAF* mutations were screened from urine and matched plasma samples. Even though the concordance achieved was low, they showed the feasibility of using urine samples for non-urogenital tract tumor mutation screening [106]. 

### 4.2. Microsatellite Instability (MSI)

The DNA mismatch repair deficiency (dMMR) causes the accumulation of a high number of DNA replication errors in DNA microsatellites. This phenome is termed as microsatellite instability (MSI) [119,134]. The frequency of dMMR/MSI-High (MSI-H) in CRC patients is 15% in early stages and 4–2% in mCRC [134]. Over the last few years, dMMR/High-MSI (MSI-H) testing has become key for all advanced CRC cancers since it is a predictive pan-tumor biomarker of immunotherapy treatment efficacy [135].

Minimally invasive detection of MSI-H from ctDNA is a promising diagnostic and treatment monitoring tool. A ddPCR assay has been developed to assess the microsatellite markers BAT-26, activin A receptor type 2A (ACVR2A), and defensin beta 105A/B (DEFB105A/B). The MSI-ddPCR assays were validated in tissue and blood samples achieving a sensitive detection of <0.1 MAF and a 100% of concordance with the most commonly used commercial kit, the pentaplex-PCR assay [119]. 

The new MSI-ddPCR assay promises to be a cost-effective, simple, and fast diagnostic tool for the detection of MSI with high clinical sensitivity. Additionally, the assay is equally compatible with solid and liquid biopsies, also including samples of cancers with low MSI frequency.

## 5. Pancreatic Cancers

Although pancreatic cancer (PC), is not very frequent, its aggressiveness implies that the ratio of cases per number of deaths is close to 1. Nowadays, it is the seventh leading cause of cancer death worldwide and it has been estimated that it will surpass breast cancer in approximately 5 years [1]. Similar to CRC, the majority of patients are diagnosed with advanced stages and only 10–15% of PC patients have localized disease at the time of diagnosis [136]. Despite different genetic mutations identified in *KRAS*, *CDKN2A*, *SMAD4,* and *TP53* genes, nearly all of them have failed to facilitate a treatment approach and patients continue receiving chemotherapy and radiotherapy depending on tumor stage [120,137]. 

Recently, the FDA approved olaparib, the first targeted therapy to increase PFS in metastatic PC patients with *BRCA* germline mutations [138].

### 5.1. KRAS

*KRAS* represents an important biomarker for PC since it appears to be mutated up to 90%. Alterations in this gene tend to be associated with reduced OS, regardless of the PC stage [139]. Several studies have evaluated the role of plasma ctDNA *KRAS* in the diagnosis, prognosis, and treatment of PC.

It has been observed how *KRAS* hotspot mutations G12A, G12C, G12D, G12R, G12S, G12V, and G13D detected via multiplex ddPCR were more represented in samples from metastatic PC patients than in locally advanced disease [120,121,122,123]. The amount of *KRAS* mutated ctDNA increases in advanced disease stages and has been significantly associated with the presence of distant organ metastasis [120]. Additionally, plasma ctDNA *KRAS* mutation incidence has been significantly associated with poor prognosis and OS [124,125,126,127]. In a locally advanced unresectable PC cohort, ctDNA *KRAS* mutation concentration was significantly lower after treatment [140]. In a similar study, researchers observed a better response to therapy in patients with whom *KRAS* ctDNA was not detectable or had disappeared within 6 months of treatment [128]. These results are in concordance with another study in which a multiplex ddPCR assay was used to detect 16 *KRAS* mutations (G12A, G12C, G12D, G12F, G12G, G12L, G12R, G12S, G12V and G13A, G13C, G13D, G13G, G13R, G13S, and G13V) (Table 4) and 7 NRAS mutations (Q61R, Q61K, Q61L, Q61H, Q61P, Q61E, and E62K) [123]. Sugimori et al. noted how *KRAS* mutations were detected at disease progression in some patients, whereas in some of them, the mutations disappeared after chemotherapy treatment. Importantly, in those patients, the mutations appeared at the same time as disease recurrence or even earlier. These findings highlight the predictive value of plasma longitudinal ctDNA monitoring for disease progression and response to treatment in PC patients [123]. 

As it has been assessed in different tumor types, bloodstream EVs represent an alternative source of ctDNA to provide information on disease evolution. In another study, ddPCR was used to assess *KRAS* hotspot mutations from EVs-derived DNA and matched cfDNA isolated from PC patients [129]. The sensitivity and specificity were 75.4% and 92.6%, respectively. Interestingly, *KRAS* mutation detection in EVs was superior to plasma cfDNA across all stages. Additionally, the observation of mutation rate in the localized pre- and post-resection cohort showed a precipitous decrease from 66% to 5%, respectively. 

### 5.2. Others

Recent studies confirmed the importance of genotyping different mutations in various genes aside from *KRAS* [141]. ddPCR was used to screen mutations previously identified via NGS in the *KRAS*, *BRCA2* (S2378X), *EGFR* (R521K), *ERBB2* (I655V, P1170A), and *KRAS* genes in a cohort of metastatic PDAC patients (NCT02017015). As expected, the *KRAS* mutation rate was 72.3%, whereas *BRCA2*, *EGFR,* and *ERBB2* were 11.7%, 13.3%, and 6.4%, respectively [142]. Other analyses associated the *ERBB2* I655V mutation with worse OS among metastatic PC patients. Further analyses are required to determine whether those patients would benefit from targeted therapy using, for example, trastuzumab. These results remark the need for target gene sequencing analysis using ctDNA-based liquid biopsy samples to better guide individualized treatments.

## 6. Clinical Trials Using ddPCR

The potential utility of ddPCR technology in clinical research is shown by the large number of clinical trials and enrolled cancer patients [143]. The growing number of biomarkers, targeted drugs, and immunotherapies have revolutionized patient treatments. However, there is an emerging need for identifying the driver mutations that are involved in treatment response, which is critical for therapeutic decision-making. In Table 5 we explore the clinical trials that are focusing on the ddPCR system to measure and quantify actionable biomarkers before, during, and after cancer treatment. Remarkably, the European Medicines Agency (EMA) includes the use of liquid biopsies for NSCLC management [144]. There are several active clinical trials recruiting NSCLC patients, in fact, most of them use the ddPCR method to evaluate the *EGFR* mutation status, indicating its high clinical significance. In the case of breast cancer, the only ongoing studies enrolled mBC patients for *ESR1* and *HER2* analyses. Among people diagnosed with mCRC, ddPCR assesses *RAS* mutations together with *MGMT* methylation for response to treatment. Thousands of volunteers would be involved in a prospective study for the detection and tracking of specific mutations in cfDNA isolated from CRC patients. 

## 7. Conclusions

Liquid biopsies are considered a good alternative and complementary tool for cancer management. The study of specific biomarkers by high throughput techniques could guide clinicians in the monitoring of disease evolution during the administration of targeted therapies. Although ddPCR has demonstrated its high sensitivity and specificity rates for detecting rare actionable mutations, further studies are required to implement it in all clinical laboratories for precision medicine.

## Figures and Tables

**Table 1:** Comprehensive comparison of liquid biopsy analysis techniques.

Technique	Sensitivity	Specificity	LoD	Advantages	Limitations	Price	References
PCR-based techniques (qRT-PCR, COLD-PCR)	29–95.7%	69.2–100%	0.1%	Rapid. No bioinformatic analysis required.	Screening of a few known mutations at a time.	Low	[6,11,17,18]
Drop-digital PCR	66.7–90%	100%	0.01%	Rapid. High sensitivity. Applicable for the detection of specific point mutations, copy-number variations, short indels, and gene fusions. No bioinformatic analysis required. Cost-effective.	Screening of a few known mutations at a time.	Medium	[6,11,15,19]
NGS-based approaches	50.9–100%	70–100%	0.1%	Molecular alteration knowledge not required Analysis of several alterations in several genes at the same time.	Expensive. Limited sensitivity. Highly time-consuming. Experienced bioinformaticians	High	[6,11,15,20,21]

LoD = limit of detection. COLD-PCR, co-amplification at lower denaturation temperature PCR. NGS = next-generation sequencing.

**Table 2:** The most relevant molecular alterations detected via ctDNA ddPCR assays and their clinical significance in NSCLC.

Disease	Oncogene	Alteration	Clinical Significance	References
NSCLC	*EGFR*	Exon 19 del L858R L861Q	Sensitivity to first-generation EGFR-TKIs	[23,24,30,31,32]
		T790M	Resistance to first- and second-generation EGFR-TKIs and sensitivity to third-generation EGFR-TKIs	[30,31,34,35,36,37,38,39]
		C797S	Resistance to third-generation EGFR-TKIs	[40,41,42,43]
	*KRAS*	G12/G13	Poor prognosis and decreased EGFR-TKIs sensitivity	[51,52,53,56,57,58,59,60,62]
		G12C	Sensitivity to sotorasib	[54,55]
	*ALK*	Mutations in the tyrosine kinase domain	G1202R, F1174C/L L1196M, G1269A, C1156Y, 1151Tins, L1152R, S1206Y, I1171T, D1203N, and V1180L mutations may confer resistance or sensitivity to the different ALK-TKIs therapies available	[67,68,69,70,71]

**Table 3:** Most relevant molecular alterations detected via ctDNA ddPCR assays and their clinical significance in BC.

Disease	Oncogene	Alteration	Clinical Significance	References
BC, advanced BC, and mBC	*BRCA1/* *BRCA2*	Point mutations, large genomic rearrangements or CNVs	Response to PARP inhibitors	[72,78,79]
	*PIK3CA*	E545K, E542K, H1047R and H1047L	Hormonal therapies resistance	[80,81,82,83]
	*ESR1*	E380Q, L536H, L536R, Y537C, Y537N (T > A), Y537N (delinsTA), Y537S and D538G	Endocrine treatment resistance	[84,86,87,88,89,90,91,92]
	*HER2*	CNV	Response to trastuzumab, pertuzumab, lapatinib, or trastuzumab emtansine	[95,96,97]

**Table 4:** The most relevant molecular alterations detected via ctDNA ddPCR assays and their clinical significance in CRC and PC.

Disease	Oncogene	Alteration	Clinical Significance	References
Colorectal Cancer	*KRAS*	G12/G13	Non-responsiveness to anti-*EGFR* monoclonal antibodies therapy such as panitumumab	[102,103,104,105,106,107,108,109,110,111,112,113,114]
	*BRAF*	V600E	Anti-*EGFR* monoclonal antibodies therapy such as cetuximab and panitumumab are not recommended unless given with a *BRAF* inhibitor such as vemurafenib or MEK and PI3K inhibitors	[105,106,109,115,116,117,118]
	*MSI*	dMMR and MSI-H	Predict response to immunotherapy	[119]
Pancreatic cancer	*KRAS*	G12/G13	Associated with poor prognosis and OS	[120,121,122,123,124,125,126,127,128,129]

**Table 5:** An overview of the ongoing clinical trials in which the ddPCR technique is used for actionable biomarker detection.

Disease.	Identifier	Aims	State	Number of Patients
NSCLC	NCT04720339	cfDNA quantification	Recruiting	250
	NCT02418234	*EGFR* T790M monitoring	Completed	314
	NCT02778854	Driver mutation detection	Recruiting	200
	NCT02279004	*BRAF*, *KRAS,* and *EGFR* mutation detection	Recruiting	680 *
	NCT03771404	Study genetic alteration during the follow-up	Recruiting	50
	NCT03265496	*EGFR* detection in solid and liquid biopsies	Active, not recruiting	117
	NCT03706625	Biomarker discovery in ctDNA and CTCs	Recruiting	170 *
	NCT03865511	*EGFR* in ctDNA	Recruiting	150
	NCT04814407	Immune-methylated signature identification	Recruiting	900
	NCT01930474	*EGFR* and ALK	Unknown	200
Advanced NSCLC	NCT03309462	*EGFR* in tissue and plasma	Completed	50
	NCT02282267	*EGFR* in plasma cfDNA	Unknown	188
	NCT02511288	Genetic profile ctDNA	Recruiting	900
	NCT02997501	*EGFR* T790M comparison between COBAS, ddPCR and NGS	Completed	167
	NCT04912687	*EGFR* mutation	Not yet recruiting	580
SCC Lung	NCT03938012	*MET* N375S, TP53	Recruiting	80
mBC	NCT02913430	*ESR1* mutation	Active, not recruiting	7
	NCT04720729	Chemotherapy monitoring ctDNA *HER2*	Recruiting	214
	NCT04480814	*PIK3CA* in ER+/HER2	Recruiting	120
	NCT03947736	ctDNA *HER2* amplification	Recruiting	200
	NCT03829306	Mechanism of Kadcyla resistance	Recruiting	50
	NCT03357120	ctDNA after neoadjuvant chemotherapy	Recruiting	180
	NCT02473120	*ESR1* mutations	Completed	104
mCRC	NCT02994888	ctDNA for cetuximab monitoring	Completed	47
	NCT03832621	*MGMT* methylation	Active	135
	NCT04554836	*RAS* mutation monitoring	Recruiting	144
	NCT03227926	*RAS* mutation monitoring	Recruiting	129
CRC	NCT04050345	*KRAS*, *NRAS*, *BRAF*, *PIK3CA*, *TP53* and *APC* detection	Recruiting	1000

* The study includes participants diagnosed with other cancer types.
